# VHZ is a novel centrosomal phosphatase associated with cell growth and human primary cancers

**DOI:** 10.1186/1476-4598-9-128

**Published:** 2010-05-28

**Authors:** Jing Ping Tang, Cheng Peow Tan, Jie Li, Md Monowarul Siddique, Ke Guo, Siew Wee Chan, Jung Eun Park, Wan Ngee Tay, Zhi Yuan Huang, Wen Cai Li, Jian Chen, Qi Zeng

**Affiliations:** 1 Institute of Molecular and Cell Biology, A*STAR (Agency for Science, Technology and Research), 61 Biopolis Drive, Proteos, Singapore 138673; 2 Neuroscience Lab, Singapore Health Service Pte Ltd. 7 Hospital Drive, Singapore 169611; 3 The First Affiliated Hospital of Zheng Zhou University, 40 Da Xue Lu, Henan, Zheng Zhou 450052, China

## Abstract

**Background:**

VHZ is a VH1-like (member Z) dual specific protein phosphatase encoded by DUSP23 gene. Some of the dual specific protein phosphatases (DSPs) play an important role in cell cycle control and have shown to be associated with carcinogenesis. Here, the expression of VHZ associated with cell growth and human cancers was investigated.

**Results:**

We generated a mouse monoclonal antibody (mAb clone#209) and rabbit polyclonal antibodies (rAb) against VHZ. We performed cell proliferation assay to learn how VHZ is associated with cell cycle by retroviral transduction to express VHZ, VHZ(C95S), and control vector in MCF-7 cells. Overexpression of VHZ [but not VHZ(C95S)] in MCF-7 cells promoted cell proliferation compared to control cells. shRNA-mediated knockdown of VHZ in MCF-7 cells showed that reduction of VHZ resulted in increased G1 but decreased S phase cell populations. Using indirect immunofluorescence, we showed that both exogenous and endogenous VHZ protein was localized at the centrosome in addition to its cytoplasmic distribution. Furthermore, using immunohistochemistry, we revealed that VHZ protein was overexpressed either in enlarged centrosomes (VHZ-centrosomal-stain) of some invasive ductal carcinomas (IDC) Stage I (8/65 cases) or in entire cytoplasm (VHZ-cytosol-stain) of invasive epithelia of some IDC Stage II/III (11/47 cases) of breast cancers examined. More importantly, upregulation of VHZ protein is also associated with numerous types of human cancer, in particular breast cancer. VHZ mAb may be useful as a reagent in clinical diagnosis for assessing VHZ positive tumors.

**Conclusions:**

We generated a VHZ-specific mAb to reveal that VHZ has a novel subcellular localization, namely the centrosome. VHZ is able to facilitate G1/S cell cycle transition in a PTP activity-dependent manner. The upregulation of its protein levels in primary human cancers supports the clinical relevance of the protein in cancers.

## Introduction

Aberrant protein tyrosine phosphorylation can result from the dysregulated expression of protein tyrosine kinases (PTKs) or protein tyrosine phosphatases (PTPs) [1]. Protein phosphorylation and dephosphorylation are major regulatory events in many cellular and pathogenic processes [2]. Considerable attention has centered on protein kinases in cancer development, however the role of protein phosphatases in cancer is still an under-explored area.

In recent years, emerging evidence indicates that members of the phosphatase of regenerating liver (PRL) subgroup of PTPs are linked to multiple human cancers [3]. The PRL-PTP family comprises three members: PRL-1, PRL-2, and PRL-3. Compelling evidence suggests that each PRL-PTP member might individually participate in the process of cancer development and metastases [1]. In particular, PRL-3 is the most thoroughly investigated member of this subgroup; and was first shown to be a metastasis-associated phosphatase that is consistently overexpressed in metastatic colorectal cancer (CRC) [4]. In an attempt to identify more PRL-PTP-related phosphatases, we used the human PRL-3 amino acid sequence to perform BLAST database searches and found VHZ that shares about 28% amino acid sequence identity with human PRL-PTPs. Indeed, VHZ was cloned and reported by three independent groups in 2004 [5-7].

There are 107 known human protein tyrosine phosphatases (PTPs) [8]. Among these PTPs, there is a class called dual-specific or VH1-like PTPs (http://www.ptphome.net/resource/resource.html[8]). The VH1-like family consists of 63 members, which comprises 19 Atypical Dual-Specific Phosphatases (DUSP), 16 Myotubularins, 11 Map Kinase Phosphatases (MKPs), 5 PTEN members, 4 CDC14 members, 3 PRLs (PRL-1, PRL-2, and PRL-3), and a few others. VHZ belongs to the atypical DUSP and has also been referred to as DUSP23, DUSP25, FLJ20442, LMW-DSP3. The HUGO accepted nomenclature for this gene is DUSP23, we have referred to DUSP23-encoded protein as VHZ since it is a member of the VH1-like phosphatase family. VHZ is expressed in many tissues and is located in both the cytosol and in nucleoli [5]. It is the smallest (calculated 16-kDa) of the catalytically active protein-tyrosine phosphatases (PTP) [5]. The crystal structure of VHZ has recently been determined at 1.93 Å resolution [9]. Despite VHZ's remarkably high degree of conservation throughout evolution with orthologues in frogs, fish, fly, and the Archaea, the physiological role of VHZ is still largely unknown since it was first cloned and identified six years ago [5-7].

VHZ and VHR belong to the same subgroup of VH1-like PTPs [8]. Since VHR has been reported to have a function in regulating cell cycle progression [10], we attempt to investigate if VHZ also plays a role in regulating cell cycle. We revealed that VHZ has an additional novel centrosomal localization and show that VHZ has a capacity to enhance G1/S phase transition. Furthermore, many dual specific protein phosphatases (DSPs), such as MKPs [11], are associated with carcinogenesis. We also investigated the expression levels of VHZ in multiple human primary cancers and showed that VHZ is overexpressed in human cancers especially in breast cancers.

## Materials and methods

### Generation of VHZ-EGFP, VHZ(C95S)-EGFP, GST-VHZ expression constructs

The human Universal Quick-clone II cDNA library (BD, Cat#637260) was used as template in the generation of VHZ cDNA. Forward primer A; 5'gcgaattcaccatgggcgtgcagccccccaacttctcc3' and reverse primer B; 5'gtggatcccgtttcgttcgctggtag 3' were used to perform PCR. The VHZ PCR fragment was then inserted into the EcoR1 and BamH1 sites of the pEGFP-N1 vector, resulting in VHZ C-terminally tagged with EGFP (VHZ-EGFP). To construct VHZ(C95S), the above forward primer A and reverse primer B together with a mid-reverse primer 5'gccaaagcccagagca***g***agtgcactcccacagc3' and a mid-forward primer 5'gcgaattcaccatgggcgtgcagccccccaacttctcc3' were used to generate catalytically inactive VHZ(C95S). The VHZ(C95S) PCR fragment was then inserted into the EcoR1 and BamH1 sites of the pEGFP-N1 vector to form VHZ(C95S)-EGFP. The VHZ PCR product was also inserted into pGEX-KG to form GST-VHZ. The authenticity of all the clones was confirmed by DNA sequencing of the coding region.

### Generation of mouse monoclonal VHZ antibody (clone #209)

We used the ClonaCell™-HY Hybridoma Cloning Kit (Stemcell Technologies Inc.) to generate VHZ hybridomas [3]. The procedures were followed according to the manufacturer's directions. Briefly: 1. Immunization of BALB/c mice with GST-VHZ fusion protein. 2. Growth of BALB/c parental myeloma cells SP2/0; 3. Preparation of BALB/c mice for spleenocytes from immunized mice 4. Fusion of spleenocytes with SP2/0 cells: 5. Selection and characterizations of the hybridoma clones. Over 500 surviving hybridoma clones were isolated and grown. By ELISA, 75 clones showed good reactions with VHZ. We searched for the best hybridoma clone that could be used for multiple applications such as ELISA, WB, IF, IHC. Finally, VHZ clone (#209) with the strongest reactivity was selected for further studies. Rabbit polyclonal anti-VHZ serum was generated (Genemed Synthesis, Inc.) by immunizing rabbits with a synthetic peptide C-RRLRPGSIETYEQEK corresponding to amino acid residues 126-140 of human VHZ. Antibodies were purified using Protein A followed by peptide affinity chromatography.

### Generation of ascetic fluids

Hybridoma cells (5 × 10^6^) were suspended in 200 μl of serum-free DMEM medium and injected with a 26-gauge needle into the peritoneal cavity. After 10 days, the mouse developed a large quantity of ascetic fluid, and the abdomen was greatly distended. The mouse was sacrificed and a small shallow was cut to open the abdominal cavity. The ascetic fluid was drawn with 10 ml syringe fitted with an 18-gauge needle. The fluid was centrifuged at 200 g for 10 min at 4°C. The supernatant fluid was collected and frozen at -70°C till further use.

### Elisa assay

GST-VHZ or pure GST antigen stocks were made in carbonated buffer (pH 9.6). Solution (100 μl) containing an indicated amount of antigen was added to each well and incubated at 4°C for overnight to coat the antigen onto the plate. The plate was then blocked with 3% bovine serum albumin in PBS containing 0.05% Tween 20 for 30 min at 37°C. The plate was then washed thrice with PBS. Culture medium or monoclonal antibodies were added to each well and incubated for 40 min at 37°C. The secondary antibody, anti-mouse IgG conjugated with horseradish peroxidase (Pierce Cat. No 31430) diluted at 1: 3000 in PBS was added to each well and incubated at 37°C for 40 min. The plate was rinsed with PBS containing 0.05% Tween-20 thrice followed by 3 washes with sterile water. The substrate, 100 μl of Turbo-TM B™ (Pierce product number 34022), was added to each well and incubated for 10 min at room temperature. The reaction was stopped by adding 100 μl of concentrated H_2_SO_4_. Absorbance was measured at 450 nm (Elisa Reader DYNATECH MR7000).

### Overexpression or shRNA-mediated knockdown of VHZ in MCF7 cells by retroviral transduction

The amphotropic Phoenix packaging cells (kindly provided by Nolan Lab at Stanford University) were transfected with the retroviral vector overexpressing VHZ, VHZ-mutant (pBABEpuro) or shRNAs in pSUPER.retro.puro vector (OligoEngine) using Effectene according to manufacturer's instruction (Roche). The short hairpin RNA sequences (shRNAs) to suppress VHZ expression were purchased from Origene Technologies (Rockville, MD). The sequences for VHZ-KD-214, VHZ-KD-215, and VHZ-KD-216 are 5'CCATGCTGGCCTGTTACCTGGTGAAGGAG3',5'CCGGCTCCATCGAGACCTATGAGCAGGAG3', and 5'AAGCAGTCTTCCAGTTCTACCAGCGAACG3' respectively. After 48 h, the retroviral supernatants were collected, filtered (0.45 μm; Millipore) and then added onto the target MCF7 cells (HTB-22), which is a human breast cancer cell line purchased from American Type Culture Collection (ATCC; Manassas, VA), in the presence of 5 μg/ml of polybrene (Sigma-Aldrich) for 6-8 h. Infection was done twice. The cells were then selected with puromycin (1 μg/ml) followed by western blot to show overexpression of VHZ or VHZ-mut in MCF7 cells. These cell clones were seeded out in 6-well plates and the numbers of cell were counted from second day to fifth day. The VHZ knockdown MCF7 cells were analyzed using bromodeoxyuridine (BrdU) incorporation assay to assess the population of cells in the S phase. Briefly, the cells were incubated with BrdU for 15 mins and the incorporated BrdU was detected by flow cytometric analysis using BrdU Flow Kit from BD Pharmingen.

### Colony formation assay

Cells (10,000) are seeded out in 6-well plates to form colonies in 2 weeks. Colonies are fixed with acetic acid and methanol (10% v/v each) for 15 minutes, stained with 0.4% crystal violet in 20% ethanol for 30 mins and rinsed with water to remove extra dye.

### FACS analyses

Cells were dissociated by trypsin treatment and washed once with PBS and fixed with 70% ethanol overnight at 4°C. The cells were then centrifuged and washed twice in 2 ml of PBS. The cell pellet was resuspended in PBS and treated with 100 μg/ml RNase A (QIAGEN). 50 μg/ml propidium iodide was added just before FACS analysis (Becton Dickinson, NJ, USA). Acquired data were analyzed by WinMDI 2.8 software to determine the percentage of cells in different cell cycle stages.

### Analysis of exogenous VHZ-EGFP subcellular localization in NRK cells

NRK cells (ATCC CRL-6509) transfected with VHZ-EGFP expressing constructs were grown on coverslips and washed once with PBSCM (PBS containing 1 mM MgCl2 and 1 mM CaCl2). Cells were then fixed with 100% methanol for 15 mins at -20°C. The methanol fixation can reduce the cytosomal VHZ to clearly reveal the centrosomal VHZ signals. After two washes with PBSCM, the cells were permeabilized for 15 mins with 0.12% Saponin in PBSCM and incubated with rabbit anti-pericentrin antibody from Covance' Inc (Princeton, NJ) for 1 hour at room temperature (RT), and then overnight at 4°C. The cells were gently washed three times with PBSCM and incubated with anti-mouse IgG conjugated with Texas Red (Sigma) for 4 hrs at RT. The VHZ-EGFP was directly visualized under fluorescence microscopy. Confocal imaging was performed (Zeiss LSM 510 image Browser).

### Analysis of endogenous VHZ in NRK, MCF-10A, and MCF-7 cells

The NRK cells, Human Mammary Epithelial MCF-10A (ATCC CRL-10317), and MCF-7 cells were grown on coverslips and washed once with PBSCM. Cells were fixed in 2.7% paraformaldehyde for 20 min at RT or 24°C. Similar steps as described in the above section were followed. Anti-γ-tubulin antibody was obtained from Sigma. Anti-pericentrin antibody was purchased from Abcam.

### Histopathologic Analyses with Immunohistochemistry (IHC)

The immunohistochemistry was performed as previously described [3]. Positive signals were detected by staining with 3,3'-diaminobenzidine chromogen (Dako, Carpinteria, CA). 80 formalin-fixed and paraffin-embedded surgical specimens of primary human breast cancer samples were collected from the archives of the Pathology Department of the Henan Medical Hospital. In addition, 32 human breast carcinoma tissues (CC08-02) were purchased from Cybrdi (Frederick, MD). Majority of the tissues were purchased from Cybrdi, Inc. (Rockville, Maryland 20850 USA: http://cybrdi.com/index.php) and include a major solid tumors tissue array (CC00-01-006); squamous cell carcinoma (CC00-01-009); lung carcinoma (CC04-01-006); colon adenocarcinoma (Grade I~III) with normal tissue controls (CC05-01-001); pancreatic carcinoma (Duct adenocarcinoma/islet cell carcinoma/mucinous carcinoma) with normal controls (CC14-01-001).

## Findings and Discussion

### Generations of VHZ mAb (clone #209)

**Figure 1:**
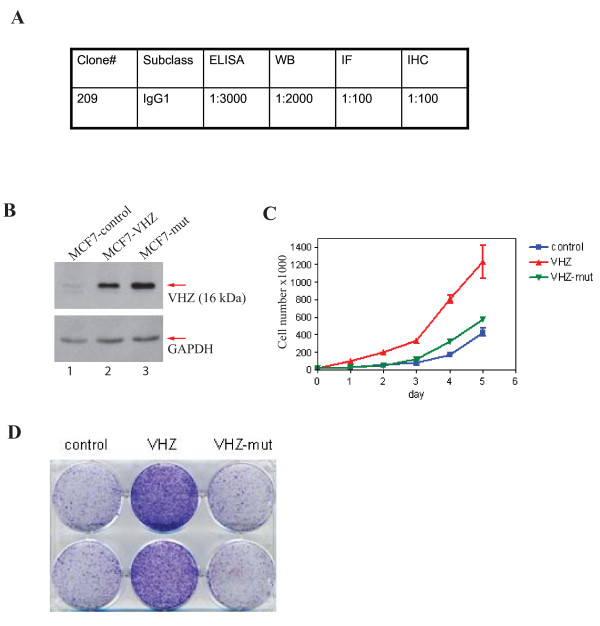
**A: Characterization of VHZ mAb (clone #209)**. The mAb secreted by clone #209 hybridoma was extensively characterized. The property and its uses (folds of dilution for ascitic fluid) in various applications were summarized. WB: Western blot; IF: immunofluorescence; IHC: immunohistochemistry. (S Figure 1 for Elisa data) B: Endogenous VHZ was detected in cells transduced with vector control (lane 1), while increased levels of VHZ protein were observed in cells transduced with VHZ (lane 2) or VHZ-mut (lane 3) expressing construct. The exogenous VHZ and VHZ-mut have identical size to endogenous VHZ protein (land 1). C: VHZ promotes cell proliferation. Cell growth was assessed for MCF7 cells transduced with control vector, VHZ or VHZ-mut. The numbers of cell (×1000) were plotted over the time of culture.VHZ-expressing cells displayed enhanced growth as compared to control and VHZ-mut cells. D: Colony forming assay. 10,000 cells as indicated were plated onto 6-well plates and grown for 2 weeks. The cells were then stained and images were taken.Cells expressing VHZ displayed enhanced ability of colony formation as compared to control and VHZ-mut expressing cells.

There is a high degree of homology within the DUSP protein family, it is therefore difficult to generate specific antibodies that do not cross-react with other DUSP proteins. ClonaCell™-HY Hybridoma Cloning Kit http://www.biowish.com/system/uploadtechN/DC22L44J9P.pdf was used to generate the VHZ hybridoma. After fusing spleenocytes derived from mouse immunized with GST-VHZ protein with SP2/0 myeloma cells, 502 surviving hybridoma clones were isolated and grown in culture. By ELISA, 75 clones showed good reactions with VHZ were subjected to evaluation by several critical tests. Only one clone (#209) was finally selected as the best since it can be used for multiple applications (ELISA, WB, IF, and IHC) (Figure 1A). The mAb secreted by clone #209 hybridoma was extensively characterized. The property and its uses (folds of dilution of ascetic fluid) in various applications were summarized. The multiple applications of the VHZ mAb were shown throughout this study (Elisa data in Additional file 1, Additional file 2). The VHZ specific antibody will be beneficial to the community of the researchers in this field.

### Overexpression of VHZ enhances cell proliferation in MCF-7 cells

To test if VHZ plays a role in cell cycle regulation, we generated three MCF-7 stable pools that express control vector, VHZ, and VHZ(C95S) respectively by retroviral transduction. Lysates of puromycin-selected cells derived from the cells were analyzed by western blot using VHZ (#clone 209) mAb (Figure 1B) to show the levels of VHZ and VHZ-mut were comparable (Figure 1B, lane 2-3) and the sizes of exogenous VHZ and VHZ-mut protein (lane 2-3) were identical to the size of endogenous VHZ (lane 1). The three stable pools were then subjected to assess for their cell proliferation (Figure 1C), the number of cells (×1000) were plotted over the time of culture. VHZ-expressing cells clearly enhanced cell proliferation (Figure 1C) and colony formation (Figure 1D) as compared to control and VHZ-mut expressing MCF7 cells.

### Knockdown of VHZ retards G1/S transition in MCF-7

**Figure 2:**
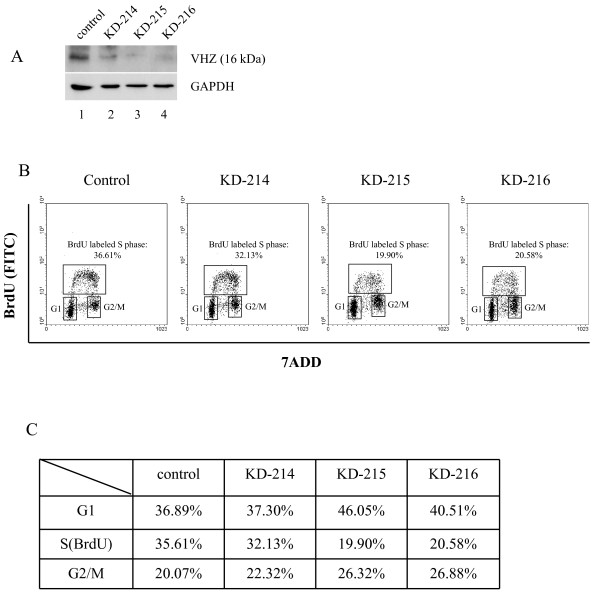
**Knockdown of VHZ in MCF7 cells inhibits G1/S transition**. **A**, the expression levels of VHZ in MCF7 cells transduced with the control (lane 1) and various shRNAs (lanes 2-4) targeting different sites of VHZ mRNA were assessed by western blot. **B**, cell cycle analysis of MCF7-control and MCF7-VHZ-KD cells that have been stained for incorporated BrdU (with FITC anti-BrdU) and total DNA levels (with 7ADD). KD-215 and KD-216 cells showed significant reduction in the levels of BrdU incorporation. **C**, Comparison of the MCF7-control cells to MCF7-VHZ-KD cells in different cell cycle phases. KD-215 and KD-216 cells have significantly increased population of cells in G1 as well as concomitant decreased population of cells in S phase.

We then carried out knockdown of VHZ in MCF-7 cells which were transduced with the control (Figure 2A, lane 1) and various shRNAs (lanes 2-4) targeting different sites of VHZ mRNA. Cell lysate was assessed by western blot to evaluate the efficiency of knockdown. The western blot (with VHZ mAb clone #209) results demonstrated that the levels of VHZ protein in KD-215 and KD-216 cells were much reduced compared to KD-214 cells (Figure 2A, lane 2). Interestingly, cells with efficient VHZ knockdown (KD-215 and KD-216 Figure 2A, lane 3-4) exhibited decrease in DNA replication as assessed by BrdU incorporation (Figure 2B), which was accompanied with increased population of cells in the G1 phase and decreased population of cells in the S phase (Figure 2C). These results indicate that knockdown of VHZ retarded cell cycle progression from G1 to S phase and are consistent with the observation that VHZ overexpression enhanced cell proliferation (Figure 1). Overall, the data support that VHZ may facilitate G1-S transition during cell cycle, and have the ability to promote cell proliferation.

### VHZ is localized at the centrosome and the cytoplasm

**Figure 3:**
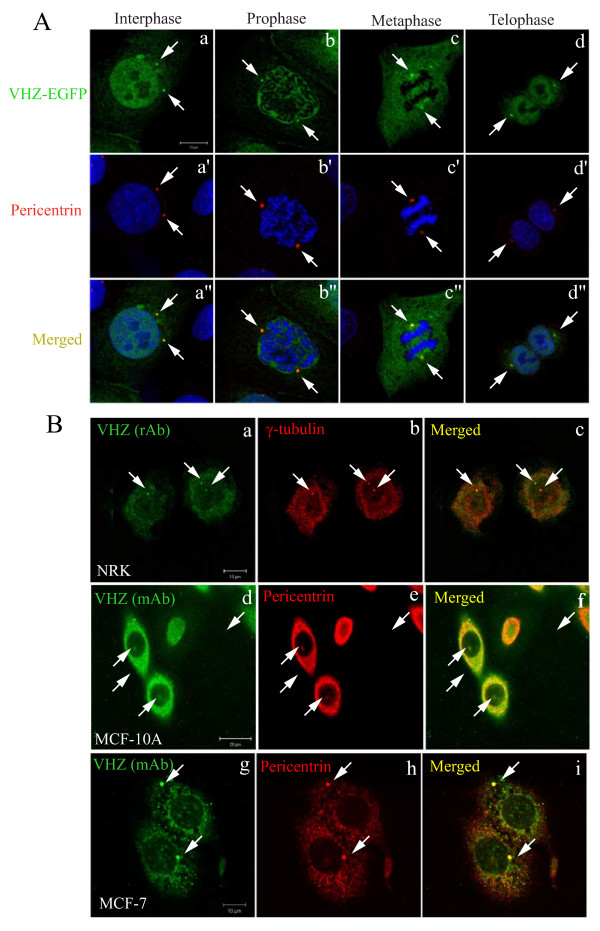
**Exogenous and endogenous VHZ are localized in the centrosome and the cytoplasm**. A. VHZ-EGFP was transfected into NRK cells and visualized in cells at various cell cycle stages: Interphase (a), Prophase (b), Metaphase (c), and Telophase (d). Pericentrin is labeled in red and nuclei are labeled with To-pro-3 iodide in blue (a'-d'). The images were merged as shown (a"-d"). Bar, 10 μm. B. Endogenous VHZ was detected in NRK (a-c, bar, 10 μm) cells by double staining with affinity-purified rabbit anti-VHZ (rAb) and mouse anti-γ-tubulin antibodies followed by anti-rabbit IgG conjugated with anti-rabbit-FITC (green) and anti-mouse IgG conjugated with anti-mouse-Texas Red. Endogenous VHZ was also detected in MCF10A and MCF-7 cells (d-f, bar, 20 μm and g-I, bar, 10 μm) by double staining with mouse anti-VHZ (mAb) and rabbit anti-pericentrin antibodies followed by anti-mouse IgG conjugated with anti-mouse-FITC (green) and anti-rabbit IgG conjugated with anti-rabbit-Texas Red.

To understand the role of VHZ in cell cycle regulation, we assessed the subcellular localization of VHZ by generating NRK cells that stably overexpress VHZ-EGFP. It is worthy to point out that truncated EGFP protein is often artificially located to the nuclei. Therefore, we observed the VHZ-EGFP localized to the nuclei of interphase, prophase, and telophase cells and appeared to redistribute to the cytoplasm of cells undergoing metaphase (Figure 3A). Importantly, the VHZ-EGFP was enriched in the centrosomes of cells at all stages of the cell cycle, as established by its co-localization with the pericentrin (a centrosomal marker) (Figure 3A). The localization of endogenous VHZ was determined by double immunofluorescence labeling with affinity-purified rabbit polyclonal anti-VHZ antibody in conjunction with mouse monoclonal (mAb) anti-γ-tubulin (another centrosomal marker) antibody. Endogenous VHZ was clearly present in the centrosomes in NRK cells where it co-localized with γ-tubulin (Figure 3B, a-c). Anti-VHZ mAb together with rabbit anti-pericentrin antibody also revealed that endogenous VHZ was co-localized to the centrosome and cytoplasm of MCF10A and MCF-7 cells (Figure 3B, d-i). The localization of VHZ to the centrosomal regions, which is of particular interest, gives some insight to the potential role of this phosphatase.

**Figure 4:**
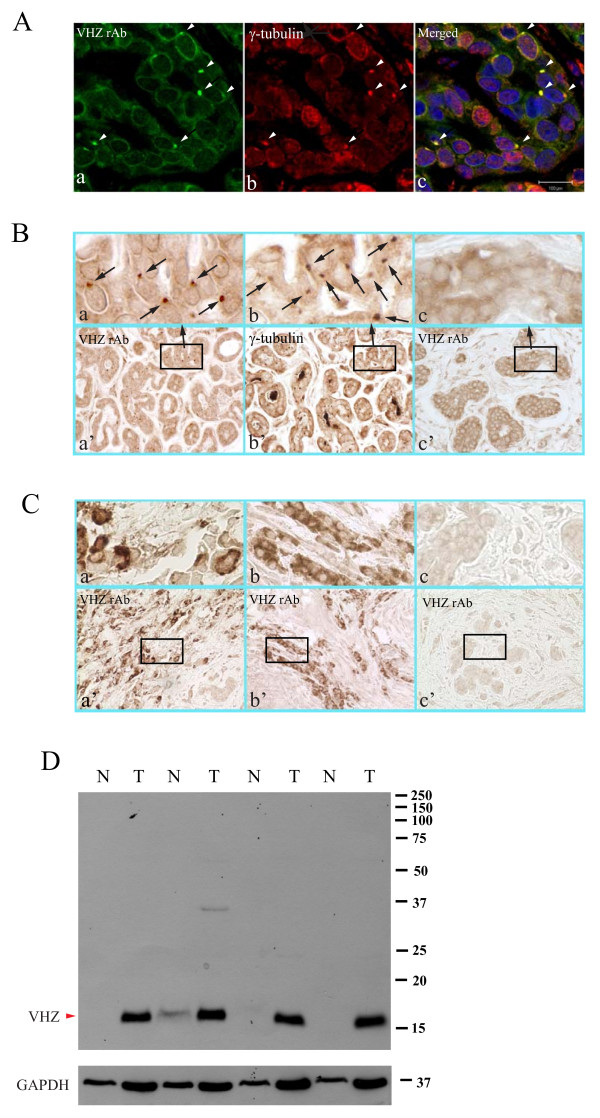
**VHZ is overexpressed in breast cancer**. Breast cancer samples were assessed by indirect double IF) and IHC for VHZ expression. **A**. Double-labeling of VHZ and γ-tubulin in the same tissue section. VHZ (c) co-localized with γ-tubulin (b) at enlarged centrosomes (white arrowheads) of cells. Image c shows merged images a and b. Bar: 100 μm. **B**. IHC detection of VHZ or γ-tubulin in two consecutive sections of the same breast cancer sample. Positive signals showed VHZ in brown. Similar centrosomal labeling patterns of VHZ (a) and γ-tubulin localization (b) were indicated by the black arrows. Three rectangular areas boxed in overview images (a'-c', magnification ×630) were further enlarged (×5) and shown in panels a-c, respectively. Panel c showed VHZ non-expressing breast cancer sample as a negative control. **C**. VHZ was overexpressed throughout the cytoplasm of dispersed epithelia in some breast cancer samples. Selected sections from different breast samples were shown in overview images (a' and b'). Three rectangular areas boxed in the overview images (a'-c', × 400) were further enlarged (×5) and shown as top panels a - c, respectively. Panel c showed one of the DUSP23-negative samples. Normal breast tissue showed no signals of VHZ protein expression (S. Figure 2A). **D**. A full western blot with 4 pairs of human breast tissues showed that VHZ protein (16 kDa) was detected in all 4 breast tumor samples indicated, but not respective matched normal tissues (except 1 with low level). T = tumor tissue, while N = normal tissue.

VHZ is overexpressed in some human breast cancers. Many human tumors show centrosomal aberrations. The centrosome is an organelle that plays a key role in cell-cycle progression and cell division. It organizes microtubule arrays throughout the cell cycle and plays a pivotal role in regulating cell division in meiotic and mitotic cells. Deregulation of the centrosome organelle is linked to human genetic diseases and cancer. Indeed, many human tumors show centrosome aberrations [12]. We hypothesized that like MKPs or PRLs; VHZ phosphatase might have similar functions in modulating the progression of some cancers. We investigated the expression levels of VHZ in 112 human breast cancer specimens and found that 8 out of 65 breast cancer samples (IDC/ILC stage I) expressed high levels of VHZ protein in the enlarged centrosome (VHZ-centrosomal-stain), as shown by double immunofluorescence with rabbit anti-VHZ and mouse anti-γ-tubulin on the same section of the cancer sample (Figure 4A) and by single staining with either rabbit anti-VHZ antibody or mouse anti-γ-tubulin antibody on two adjacent sections of the same sample (Figure 4B, a-b). In addition to VHZ-centrosomal-staining, we also observed a VHZ-cytoplasmic-staining pattern in a different subset of breast cancer samples that were diagnosed as IDC Stage II/III. 11 out of 47 of this type of breast cancer samples showed high levels of VHZ protein distributed throughout the cytoplasm of epithelial tumor cells that displayed a fibroblast-like morphology (Figure 4C a-b). In the latter, it is possible that a fair amount of VHZ might still be centrosomal but the staining could be masked by the increased cytoplasmic staining in these tumor cells. Tumor cells infiltrate the surrounding tissue matrices in diverse patterns including both individual- and collective-cell-migration strategies [13,14]. In our study, we showed that there were both individual-VHZ- (Figure 4C, a) and collective-VHZ-cytosol-positive cells (Figure 4C, b). Mouse VHZ mAb was used to reconfirm some of the 112 breast cancer specimens. Both rabbit and mouse VHZ antibodies show similar results. These phenomena might represent a relatively early onset of local invasion within microenvironments *in vivo*. Hoverer, by IHC, 24 normal breast tissues used as control in human breast carcinoma tissues (CC08-02) purchased from Cybrdi (Frederick, MD) were shown to be all negative for VHZ. One representative image was shown in S. Figure 2A. Although the precise role that VHZ plays in tumor progression or cancer cell migration is not known, our data indicate that overexpression of VHZ or its elevated activity might be a crucial early event for local invasion. We then tested if VHZ could play a role in triggering cancer cell migration. To study cell mobility driven by VHZ, MCF-7 cells expressing VHZ-EGFP, or VHZ(C95S)-EGFP were examined for cell migratory properties. As migration of MCF-7 cells was difficult to measure using the conventional wound-healing or Transwell chamber assays, we used an alterative 'Inverted Coverslip' assay previously described [15]. As shown (S. Figure 2B a' white arrows), MCF7-VHZ cells migrated out from the coverslip, while MCF-VHZ(C95S) cells remain within the coverslip (S. Figure 2B b' white arrows). The results suggest that VHZ is able to promote cell motility. Most importantly, we obtained 4 pairs of human samples (breast cancers matched with their respective normal breast tissues) from 4 individuals to compare the expression of VHZ expression levels, all 4 breast tumors showed robust VHZ upregulation while 3 normal breast tissues showed no expression with 1 showed low expression (Figure 4D), indicating that VHZ is specifically overexpressed in these breast cancers examined. This result also further confirmed the specificity of VHZ mAb in recognizing endogenous protein. The data suggest that VHZ may potentially be a useful therapeutic target for breast cancer.

### VHZ is elevated in a significant fraction of several types of human cancers

**Figure 5:**
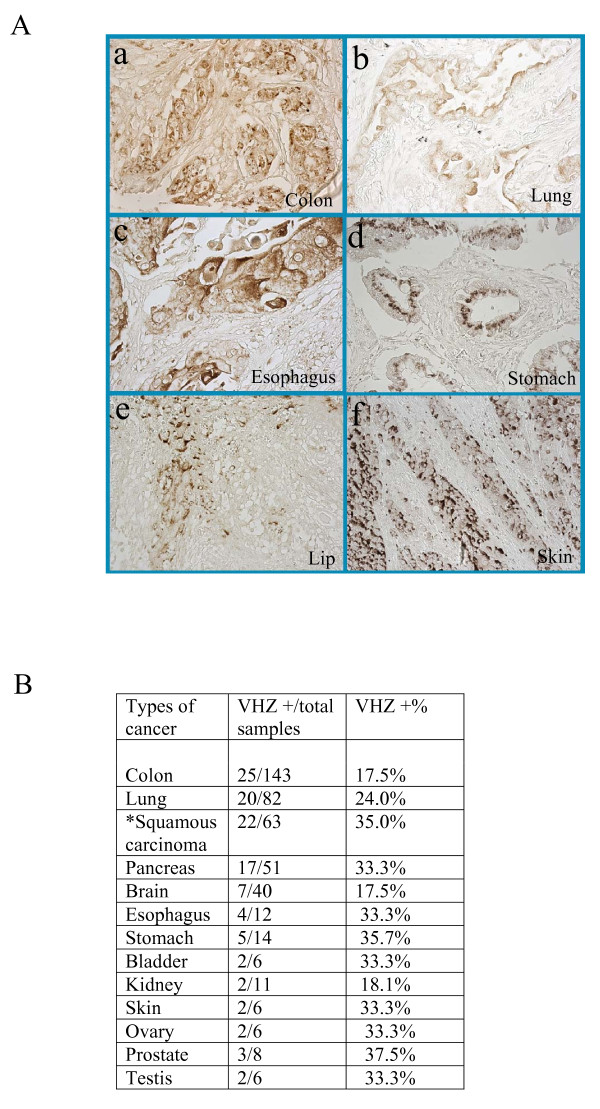
**VHZ is elevated in a significant fraction of several types of human cancers**. **A**. VHZ-positive signals (DAB chromogen in brown) were mainly detected at the plasma membrane, cytosol, and the Golgi-like sub-cellular structures in the cytoplasm. VHZ overexpression was closely associated with squamous cell carcinoma in the lung, bladder, esophagus, skin, lip, larynx, vulva, cervix, and penis etc. Selected VHZ-positive cancer samples from the colon (a, ×400), lung (b, ×400), esophagus (c, ×400), stomach (d, ×400), lip (e, ×200), and skin (f, ×200) were shown. **B**. VHZ protein expresses in multiple human cancers. Percentages of VHZ positive cancers were summarized from a total of 448 human cancer samples. VHZ enhanced MCF-7 cells migration (S. Figure 2B). * Squamous cell carcinoma such as lip, larynx, vulva, cervix, penis etc.

In addition to 112 breast cancers examined with rabbit VHZ antibodies, we also reconfirmed the data with VHZ mAb (clone #209) and obtained similar results; we further used the specific VHZ mAb (clone #209) to examine 448 multiple human cancers (Figure 5) to determine the expression of VHZ in other types of human cancers by immunohistochemistry (IHC) [3]. VHZ level was elevated in 17.5% (25 out of 143 cases) of colon cancers, 24.0% (20 out of 82 cases) of lung cancers, 35.0% (22 out of 63 cases) of squamous carcinoma (such as the lip, pharynx, vulva, cervix, and penis etc), 33.3% (17 out of 51 cases) of pancreatic cancers, 17.5% (7 out of 40 cases) of brain cancers, 33.3% (4 out of 12 cases) of esophageal cancers, 35.7% (5 out of 14 cases) of stomach cancers, 2 out of 6 cases of bladder cancers, 2 out of 11 cases of kidney cancers, 2 out of 6 cases of skin cancers, 2 out of 6 cases of ovary cancers, 3 out of 8 cases of prostate cancers, and 2 out of 6 cases of testes cancers. These observations imply that VHZ overexpression may be associated with the development or progression of various human cancers, although the functional and clinical implications need to be addressed by future experiments. Our VHZ mAb will be useful as a reagent in clinical diagnosis for VHZ expression and for research community in the field of DUSP protein family.

Our findings reveal new insight into VHZ centrosomal localization and elucidate its role in promoting cell proliferation by participating in G1-S transition. The observed overexpression of VHZ in human cancers especially in breast cancer indicates that VHZ may have a proliferative role in human cancer. Further investigation of this phosphatase will help to increase understanding of the molecular mechanism responsible for its pro-proliferative role. The mAb developed and characterized here will be an important reagent to the scientific community for further studies on VHZ phosphatase.

## Abbreviations

VHZ: VH1-like member Z; PRL: phosphatase of regenerating liver; PTP: protein tyrosine phosphatase; WB: western blot; IF: immunofluorescence; IHC: immunohistochemistry; FACS: fluorescence-activated cell sorter; EGFP: enhanced green fluorescent protein; MCF10A: human breast epithelia cell line; MCF-7: human breast cancer cell line; NRK: normal rat kidney cell line; IDC: invasive ductal carcinoma.

## Competing interests

The authors declare that they have no competing interests.

## Authors' contributions

QZ designed and drafted the manuscript. JPT did molecular cloning, transfection, IF, CPT, MMS, and HZY carried out PCR and western blot, LJ generated mAb (#209) against VHZ and performed IHC, JEP and SWC carried out the experiments for cell proliferation, knockdown, and FACS. GK, LWC, and CJ analyzed data from IHC on human cancer samples. WNT helped in discussion. All authors approved the final manuscript.

## Supplementary Material

Additional file 1 **VHZ (clone #209) mAb and Rabbit polyclonal antibodies are specific against VHZ antigen by Elisa.** To assess the specificity of VHZ antibodies, 96-well plate was coated with indicated GST or GST-VHZ antigen (10 ng). Both antibodies showed 9-11 times more reactivity to 10 ng of GST-VHZ antigen than to pure GST antigens. Click here for file

Additional file 2 **A**.** VHZ is not expressed in normal human breast tissue by IHC.B**. Overexpression of VHZ in MCF-7 cells enhances cell migration. Cell motility was assessed by growing MCF-7 cells overexpressing VHZ-EGFP or VHZ (C95S)-EGFP to form a confluent monolayer on a coverslip. The cell-coated coverslip was then inverted with the cell side down onto a fresh culture dish. Images were taken at 0 and 48 h of the MCF-7-VHZ-EGFP cells (a, a') and the MCF-7-VHZ (C95S)-EGFP (b, b'). Panel a' shows MCF-7-VHZ-EGFP cells moving out (indicated by the arrows) from underneath the coverslip. Immunofluorescent images (a, b). Phase-contrast images (a', b' magnification ×200). Click here for file
