# Supplementary figures and images for: VHZ is a novel centrosomal phosphatase associated with cell growth and human primary cancers
Source: Mol Cancer. 2010 May 28;9:128. doi: 10.1186/1476-4598-9-128 (PMC2893100; doi:10.1186/1476-4598-9-128)

**A**

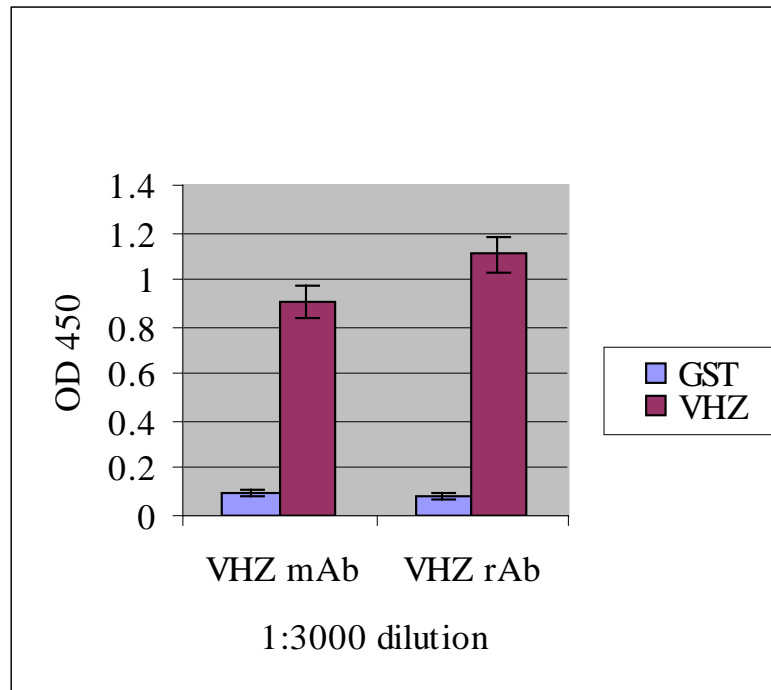

Supplement: Additional file 1 — VHZ (clone #209) mAb and Rabbit polyclonal antibodies are specific against VHZ antigen by Elisa. To assess the specificity of VHZ antibodies, 96-well plate was coated with indicated GST or GST-VHZ antigen (10 ng). Both antibodies showed 9-11 times more reactivity to 10 ng of GST-VHZ antigen than to pure GST antigens. [file 1476-4598-9-128-S1.PDF]

**A**

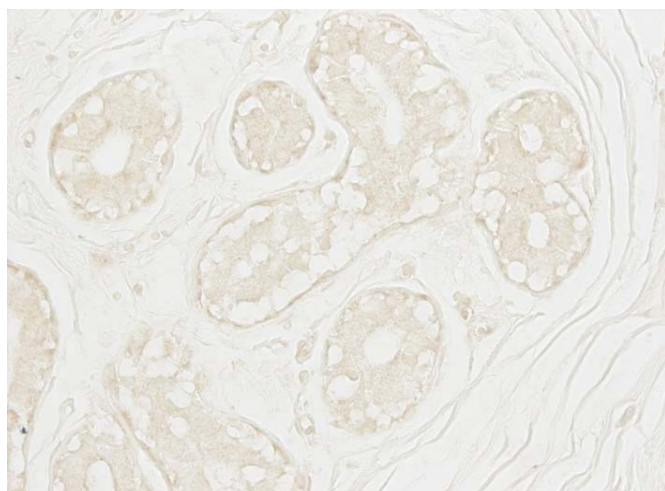

**B**

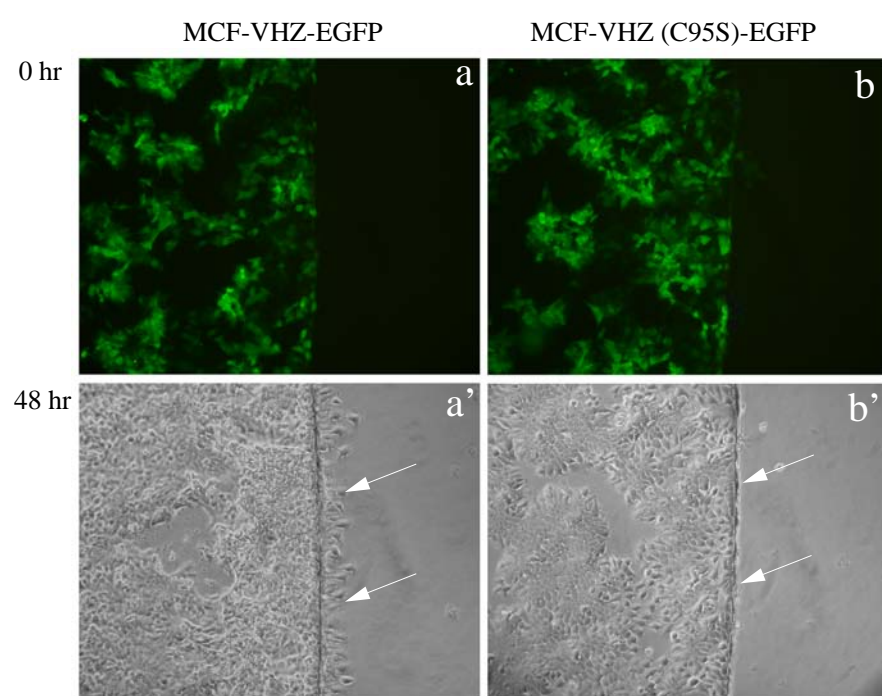

Supplement: Additional file 2 — A. VHZ is not expressed in normal human breast tissue by IHC.B. Overexpression of VHZ in MCF-7 cells enhances cell migration. Cell motility was assessed by growing MCF-7 cells overexpressing VHZ-EGFP or VHZ (C95S)-EGFP to form a confluent monolayer on a coverslip. The cell-coated coverslip was then inverted with the cell side down onto a fresh culture dish. Images were taken at 0 and 48 h of the MCF-7-VHZ-EGFP cells (a, a') and the MCF-7-VHZ (C95S)-EGFP (b, b'). Panel a' shows MCF-7-VHZ-EGFP cells moving out (indicated by the arrows) from underneath the coverslip. Immunofluorescent images (a, b). Phase-contrast images (a', b' magnification ×200). [file 1476-4598-9-128-S2.PDF]
